# Supplementary material for: Maternal Cardiovascular Risk Assessment 3-to-11 Years Postpartum in Relation to Previous Occurrence of Pregnancy-Related Complications
Source: J Clin Med. 2019 Apr 20;8(4):544. doi: 10.3390/jcm8040544 (PMC6517910; doi:10.3390/jcm8040544)
Supplement: Supplementary file 1 [file jcm-08-00544-s001.pdf]

Supplementary Material

Table S1. Impact of GH or PE history on maternal cardiovascular risk – complete overview (ANOVA and ANCOVA analyses).

		**NTP (*n* = 90)**	**FGR (*n* = 34)**	**GH (*n* = 50)**	**PE (*n* = 102)**	**Diagnostic Groups (Normal vs Diseased)**	**p Value (ANOVA, ANCOVA)**
Serum total cholesterol (mmol/L)	Unadjusted data	5.040 (0.087)	5.111 (0.141)	5.289 (0.117)	5.165 (0.081)	-	-
	Adjusted data	5.184 (0.092)[A]	5.205 (0.142)[A]	5.125 (0.121)[A]	5.104 (0.081)[A]	-	-
Serum HDL cholesterol, (mmol/L)	Unadjusted data	1.606 (0.031)	1.547 (0.051)	1.480 (0.043)	1.530 (0.029)	-	-
	Adjusted data	1.606 (0.034)[A]	1.528 (0.054)[A]	1.484 (0.046)[A]	1.537 (0.031)[A]	-	-
Serum LDL cholesterol (mmol/L)	Unadjusted data	3.119 (0.072)	3.239 (0.117)	3.439 (0.098)	3.277 (0.068)	-	-
	Adjusted data	3.210 (0.078)[A]	3.321 (0.121)[A]	3,319 (0.103)[A]	3.233 (0.069)[A]	-	-
Serum triglycerides (mmol/L)	Unadjusted data	0.847 (0.051)	0.972 (0.083)	1.023 (0.060)	0.984 (0.048)	-	-
	Adjusted data	0.897 (0.055)[A]	1.029 (0.086)[A]	0.966 (0.073)[A]	0.966 (0.049)[A]	-	-
Serum Lp(a) (nmol/L)	Unadjusted data	36.449 (7.172)	44.792 (11.605)	31.923 (9.966)	61.197 (6.733)	-	-
	Adjusted data	39.432 (7.676)[A]	45.008 (11.902)[A]	26.954 (10,129)[A]	63.489 (6.859)[A]	-	-
Serum CRP (mg/L)	Unadjusted data	3.395 (0.860)	5.314 (1.392)	2.485 (1.159)	2.903 (0.803)	-	-
	Adjusted data	3.602 (0.938)[A]	5.638 (1.456)[A]	2.482 (1.236)[A]	2.636 (0.833)[A]	-	-
Plasma homocysteine (μmol/l)	Unadjusted data	10.917 (0.302)	11.486 (0.496)	10.802 (0.407)	11.383 (0.283)	-	-
	Adjusted data	10.941 (0.332)[A]	11.565 (0.523)[A]	10.735 (0.438)[A]	11.456 (0.296)[A]	-	-
Serum uric acid (μmol/L)	Unadjusted data	248.044 (6.212)	278.529 (10.051)	286.081 (8.372)	276.792 (5.832)	NTP vs ↑FGR<br>**NTP vs ↑GH**<br>**NTP vs ↑PE**	*P* = 0.094<br>***p* = 0.004**<br>***p* = 0.005**
	Adjusted data	257.163 (6.629)[A]	280.374 (10.296)[A]	275.486 (8.740)[A]	274.948 (5.920)[A]	-	-
BMI	Unadjusted data	23.100 (0.517)	23.928 (0.941)	27.228 (0.694)	25.946 (0.486)	**NTP vs ↑GH**<br>**NTP vs ↑PE**	***p* < 0.001**<br>***p* < 0.001**
	Adjusted data	23.138 (0.525)[A]	24.529 (0.860)[A]	26.987 (0.694)[A]	25.945 (0.490)[A]	**NTP vs ↑GH**<br>**NTP vs ↑PE**	***p* = 0.002**<br>***p* = 0.001**
Waist circumference (cm)	Unadjusted data	76.605 (1.277)	78.264 (2.078)	86.770 (1.713)	83.852 (1.199)	**NTP vs ↑GH**<br>**NTP vs ↑PE**	***p* < 0.001**<br>***p* < 0.001**
	Adjusted data	76.891 (1.298)[A]	79.546 (2.128)[A]	86.118 (1.716)[A]	83.624 (1.212)[A]	**NTP vs ↑GH**	***p* = 0.001**

						**NTP vs ↑PE**	***p* = 0.001**
SBP<br>(mmHg)	Unadjusted data	112.911 (1.316)	118.088 (2.142)	129.580 (1.766)	123.656 (1.236)	**NTP vs ↑GH**<br>**NTP vs ↑PE**	***p* < 0.001**<br>***p* < 0.001**
	Adjusted data	114.895 (1.286)[A]	118.325 (2.058)[A]	127.517 (1.683)[A]	122.642 (1.178)[A]	**NTP vs ↑GH**<br>**NTP vs ↑PE**	***p* < 0.001**<br>***p* = 0.029**
DBP<br>(mmHg)	Unadjusted data	72.100 (1.000)	77.058 (1.627)	82.940 (1.342)	79.490 (0.939)	**NTP vs ↑GH**<br>**NTP vs ↑PE**	***p* < 0.001**<br>***p* < 0.001**
	Adjusted data	73.898 (0.977)[A]	77.091 (1.564)[A]	81.295 (1.279)[A]	78.737 (0.895)[A]	**NTP vs ↑GH**<br>**NTP vs ↑PE**	***p* < 0.001**<br>***p* = 0.002**
Heart rate<br>(bpm)	Unadjusted data	71.644 (1.079)	73.969 (1.782)	76.300 (1.447)	72.627 (1.013)	NTP vs ↑GH	*p* = 0.062
	Adjusted data	71.705 (1.141)[A]	74.294 (1.861)[A]	76.262 (1.497)[A]	72.670 (1.048)[A]	NTP vs ↑GH	*p* = 0.119
Relative QRISK®2 risk score	Unadjusted data	0.920 (0.134)	1.379 (0.216)	1.966 (0.178)	1.617 (0.125)	**NTP vs ↑GH**<br>**NTP vs ↑PE**	***p* < 0.001**<br>***p* < 0.001**
	Adjusted data	0.865 (0.118)[A]	1.420 (0.195)[A]	1.984 (0.155)[A]	1.578 (0.111)[A]	**NTP vs ↑GH**<br>**NTP vs ↑PE**	***p* < 0.001**<br>***p* < 0.001**

Data are expressed as mean (SE; standard error). Analysis of variance (ANOVA) was used for unadjusted data and Analysis of covariance (ANCOVA) for adjusted data. The significance level was established at *p*-value of $p < 0.05$ (Bonferroni corrected p-values). [A] Adjusted for potential covariates, where appropriate, including current maternal age, BMI, parity, the time after the birth (in months), the oral contraceptive use status (ex-user, current user, non-user), the smoking status (ex-smoker, smoker, non-smoker), the average values of systolic and diastolic blood pressures, and hypertension on the treatment (yes, no). FGR, fetal growth restriction; GH, gestational hypertension; PE, preeclampsia; NTP, normotensive term pregnancies; HDL, high-density lipoprotein; LDL, low-density lipoprotein; Lp(a), lipoprotein a; CRP, C-reactive protein; BMI, body mass index; SBP, systolic blood pressure; DBP, diastolic blood pressure; bpm, beats per minute.

Table S2. Impact of GH or PE history on maternal cardiovascular risk – complete overview (ROC curve analysis).

		Diagnostic Groups (Normal vs Diseased)	ROC Curve Parameters	Sensitivity at 10% FPR	Sensitivity and Specificity When Critical Values Are Exceeded
Serum total cholesterol (mmol/L)	Unadjusted data	-	-	-	-
	Adjusted data	-	-	-	-
Serum HDL cholesterol (mmol/L)	Unadjusted data	-	-	-	-
	Adjusted data	-	-	-	-
Serum LDL cholesterol (mmol/L)	Unadjusted data	-	-	-	-
	Adjusted data	-	-	-	-
Serum triglycerides (mmol/L)	Unadjusted data	-	-	-	-
	Adjusted data	-	-	-	-
Serum Lp(a) (nmol/L)	Unadjusted data	-	-	-	-
	Adjusted data	-	-	-	-
Serum CRP (mg/L)	Unadjusted data	-	-	-	-
	Adjusted data	-	-	-	-
Plasma homocysteine (μmol/L)	Unadjusted data	-	-	-	-
	Adjusted data	-	-	-	-
Serum uric acid (μmol/L)	Unadjusted data	NTP vs FGR	AUC 0.615, $p = 0.056$	32.35% Criterion > 308.5 μmol/l	26.46% sensitivity at 97.5% specificity Criterion > 340.55 μmol/l (hyperuricemia)
		NTP vs GH	AUC 0.670, $p < 0.001$	36.53% Criterion > 308.5 μmol/l	14.29% sensitivity at 97.5% specificity Criterion > 340.55 μmol/l (hyperuricemia)
		NTP vs PE	AUC 0.644, $p < 0.001$	28.51% Criterion > 311.2 μmol/l	13.09% sensitivity at 97.5 % specificity Criterion >340.775 μmol/l (hyperuricemia)
	Adjusted data	-	-	-	-
BMI	Unadjusted data	NTP vs GH	AUC 0.738, $p < 0.001$	42.00%	20.0% sensitivity at 97.5% specificity

				Criterion > 27.78<br>(overweight)	Criterion > 31.02<br>(obese class I, moderately obese)
		NTP vs PE	AUC 0.670, *p* < 0.001	27.45%<br>Criterion > 27.78<br>(overweight)	18.63% sensitivity at 97.5% specificity<br>Criterion > 31.02<br>(obese class I, moderately obese)
	Adjusted data	NTP vs GH	AUC 0.899, *p* < 0.001	74.00%	-
		NTP vs PE	AUC 0.791, *p* < 0.001	53.06%	-
Waist circumference<br>(cm)	Unadjusted data	NTP vs GH	AUC 0.743, *p* < 0.001	42.00%<br>Criterion > 87 cm	36.0% sensitivity at 91.11% specificity<br>Criterion > 88cm<br>(obese, high cardiovascular risk)
		NTP vs PE	AUC 0.688, *p* < 0.001	32.35%<br>Criterion >87 cm	29.41% sensitivity at 91.11% specificity<br>Criterion > 88cm<br>(obese, high cardiovascular risk)
	Adjusted data	NTP vs GH	AUC 0.902, *p* < 0.001	70.00%	-
		NTP vs PE	AUC 0.796, *p* < 0.001	54.08%	-
SBP<br>(mmHg)	Unadjusted data	NTP vs GH	AUC 0.843, *p* < 0.001	54.00%<br>Criterion > 123.4 mmHg<br>(prehypertension)	18.00% sensitivity at 100.0% specificity<br>Criterion > 141 mmHg<br>(hypertension)
		NTP vs PE	AUC 0.750, *p* < 0.001	46.86%<br>Criterion > 123.4 mmHg<br>(prehypertension)	10.78% sensitivity at 100.0% specificity<br>Criterion > 141 mm Hg<br>(hypertension)
	Adjusted data	NTP vs GH	AUC 0.822, *p* < 0.001	62.00%	-
		NTP vs PE	AUC 0.754, *p* < 0.001	51.10%	-
DBP<br>(mmHg)	Unadjusted data	NTP vs GH	AUC 0.794, *p* < 0.001	51.00%<br>Criterion > 80.5 mmHg<br>(prehypertension)	20.0% sensitivity at 100.0% specificity<br>Criterion > 91 mmHg<br>(hypertension)
		NTP vs PE	AUC 0.714, *p* < 0.001	39.71%<br>Criterion > 80.5 mmHg<br>(prehypertension)	12.75% sensitivity at 100.0% specificity<br>Criterion > 91 mmHg<br>(hypertension)
	Adjusted data	NTP vs GH	AUC 0.875, *p* < 0.001	60.00%	-

		NTP vs PE	AUC 0.778, $p < 0.001$	47.96%	-
Heart rate (bpm)	Unadjusted data	NTP vs GH	AUC 0.619, $p = 0.017$	18.00% Criterion > 84 bpm	4.0% sensitivity at 100.0% specificity Criterion > 107 bpm (tachycardia)
	Adjusted data	NTP vs GH	AUC 0.833, $p < 0.001$	54.00%	-
Relative QRISK®2 risk score	Unadjusted data	NTP vs GH	AUC 0.789, $p < 0.001$	30.00% Criterion > 1.60	18.0% sensitivity at 100.0% specificity criterion > 2.9
		NTP vs PE	AUC 0.711, $p < 0.001$	26.37% Criterion > 1.60	12.75% sensitivity at 100.0% specificity Criterion > 2.9
	Adjusted data	NTP vs GH	AUC 0.894, $p < 0.001$	74.00%	-
		NTP vs PE	AUC 0.788, $p < 0.001$	55.10%	-

The unadjusted and adjusted receivers operating characteristic (ROC) curves were constructed to calculate the area under the curve (AUC) and the best cut-off points for particular studied parameters or biomarkers were used in order to calculate the respective sensitivity at 90.0% specificity (MedCalc Software bvba, Ostend, Belgium). We also reported for unadjusted data the information showing the sensitivity at criterions exceeding the critical values (number of cases exceeding the critical values). Data were adjusted for potential covariates, where appropriate, including current maternal age, BMI, parity, the time after the birth (in months), the oral contraceptive use status (ex-user, current user, non-user), the smoking status (ex-smoker, smoker, non-smoker), the average values of systolic and diastolic blood pressures, and hypertension on the treatment (yes, no). The significance level was established at *p*-value of $p < 0.05$. FGR, fetal growth restriction; GH, gestational hypertension; PE, preeclampsia; NTP, normotensive term pregnancies; HDL, high-density lipoprotein; LDL, low-density lipoprotein; Lp(a), lipoprotein a; CRP, C-reactive protein; BMI, body mass index; SBP, systolic blood pressure; DBP, diastolic blood pressure; bpm, beats per minute; AUC, area under curve; FPR, false positive rate.

Table S3. Impact of severity of PE on maternal cardiovascular risk – complete overview (ANOVA and ANCOVA analyses).

		NTP (n = 90)	PE w/o SF (n = 25)	PE w/SF (n = 77)	Diagnostic Groups (Normal vs Diseased)	p Value (ANOVA, ANCOVA)
Serum total cholesterol (mmol/L)	Unadjusted data	5.040 (0.081)	5.145 (0.154)	5.171 (0.088)	-	-
	Adjusted data	5.124 (0.086)[A]	5.106 (0.156)[A]	5.107 (0.091)[A]	-	-
Serum HDL cholesterol (mmol/L)	Unadjusted data	1.606 (0.031)	1.497 (0.059)	1.540 (0.034)	-	-
	Adjusted data	1.578 (0.032)[A]	1.547 (0.058)[A]	1.565 (0.034)[A]	-	-
Serum LDL cholesterol (mmol/L)	Unadjusted data	3.119 (0.069)	3.303 (0.131)	3.268 (0.074)	-	-
	Adjusted data	3.208 (0.072)[A]	3.219 (0.132)[A]	3.193 (0.077)[A]	-	-
Serum triglycerides (mmol/L)	Unadjusted data	0.847 (0.050)	0.942 (0.095)	0.998 (0.054)	-	-
	Adjusted data	0.917 (0.052)[A]	0.877 (0.095)[A]	0.951 (0.055)[A]	-	-
Serum Lp(a) (nmol/L)	Unadjusted data	10.917 (0.310)	11.793 (0.586)	11.249 (0.336)	-	-
	Adjusted data	10.720 (0.336)[A]	12.047 (0.614)[A]	11.546 (0.360)[A]	-	-
Serum CRP (mg/L)	Unadjusted data	3.395 (0.870)	2.836 (1.642)	2.926 (0.936)	-	-
	Adjusted data	4.005 (0.944)[A]	2.877 (1.722)[A]	2.100 (1.004)[A]	-	-
Plasma homocysteine (μmol/L)	Unadjusted data	10.917 (0.310)	11.793 (0.586)	11.249 (0.336)	-	-
	Adjusted data	10.720 (0.336)[A]	12.047 (0.614)[A]	11.546 (0.360)[A]	-	-
Serum uric acid (μmol/L)	Unadjusted data	248.044 (5.649)	275.880 (10.659)	277.092 (6.113)	**NTP vs ↑PE w/SF**	**p= 0.001**
	Adjusted data	258.099 (5.666)[A]	267.963 (10.349)[A]	269.550 (6.077)[A]	-	-
BMI	Unadjusted data	23.100 (0.480)	26.037 (0.912)	25.916 (0.519)	**NTP vs ↑PE w/o SF**<br>**NTP vs ↑PE w/SF**	**p = 0.021**<br>**p < 0.001**
	Adjusted data	23.518 (0.479)[A]	26.300 (0.883)[A]	25.378 (0.518)[A]	**NTP vs ↑PE w/o SF**<br>**NTP vs ↑PE w/SF**	**p = 0.021**<br>**p = 0.015**
Waist circumference (cm)	Unadjusted data	76.605 (1.191)	83.480 (2.260)	83.974 (1.287)	**NTP vs ↑PE w/o SF**<br>**NTP vs ↑PE w/SF**	**p = 0.010**<br>**p < 0.001**
	Adjusted data	77.796 (1.206)[A]	83.607 (2.225)[A]	82.416 (1.304)[A]	**NTP vs ↑PE w/o SF**<br>**NTP vs ↑PE w/SF**	**p = 0.029**<br>**p = 0.013**
SBP	Unadjusted data	112.911(1.203)	122.160 (2.283)	124.142 (1.301)	**NTP vs ↑PE w/o SF**	**p = 0.001**

(mmHg)					**NTP vs ↑PE w/SF**	***p* < 0.001**
	Adjusted data	116.949 (0.743)[A]	120.994 (1.379)[A]	119.791 (0.810)[A]	**NTP vs ↑PE w/o SF**<br>**NTP vs ↑PE w/SF**	***p* = 0.055**<br>***p* = 0.121**
DBP (mmHg)	Unadjusted data	72.100 (0.934)	77.520 (1.773)	80.129 (1.010)	**NTP vs ↑PE w/o SF**<br>**NTP vs ↑PE w/SF**	***p* = 0.014**<br>***p* < 0.001**
	Adjusted data	72.896 (0.948)[A]	77.841 (1.810)[A]	79.376 (1.037)[A]	**NTP vs ↑PE w/o SF**<br>**NTP vs ↑PE w/SF**	***p* = 0.036**<br>***p* < 0.001**
Heart rate (bpm)	Unadjusted data	71.644 (1.020)	74.680 (1.936)	71.961 (1.103)	-	-
	Adjusted data	72.317 (1.067)[A]	75.242 (1.960)[A]	71.124 (1.144)[A]	-	-
Relative QRISK®2 risk score	Unadjusted data	0.920 (0.116)	1.228 (0.220)	1.744 (0.125)	**NTP vs ↑PE w/o SF**<br>**NTP vs ↑PE w/SF**	***p*= 0.008**<br>***p*< 0.001**
	Adjusted data	1.060 (0.098)[A]	1.201 (0.182)[A]	1.553 (0.106)[A]	**NTP vs ↑PE w/o SF**<br>**NTP vs ↑PE w/SF**	***p*= 0.003**<br>***p*< 0.001**

Data are expressed as mean (SE; standard error). Analysis of variance (ANOVA) was used for unadjusted data and Analysis of covariance (ANCOVA) for adjusted data. The significance level was established at *p*-value of $p < 0.05$ (Bonferroni corrected p-values). [A] Adjusted for potential covariates, where appropriate, including current maternal age, BMI, parity, the time after the birth (in months), the oral contraceptive use status (ex-user, current user, non-user), the smoking status (ex-smoker, smoker, non-smoker), the average values of systolic and diastolic blood pressures, and hypertension on the treatment (yes, no). PE w/o SF, preeclampsia without severe features; PE w/SF, preeclampsia with severe features; NTP, normotensive term pregnancies; HDL, high-density lipoprotein; LDL, low-density lipoprotein; Lp(a), lipoprotein a; CRP, C-reactive protein; BMI, body mass index; SBP, systolic blood pressure; DBP, diastolic blood pressure; bpm, beats per minute.

Table S4. Impact of severity of PE on maternal cardiovascular risk - complete overview (ROC curve analysis).

		**Diagnostic Groups (Normal vs Diseased)**	**ROC Curve Parameters**	**Sensitivity at 10% FPR**	**Sensitivity and Specificity When Critical Values Are Exceeded**
Serum total cholesterol (mmol/L)	Unadjusted data	-	-	-	-
	Adjusted data	-	-	-	-
Serum HDL cholesterol, (mmol/L)	Unadjusted data	-	-	-	-
	Adjusted data	-	-	-	-
Serum LDL cholesterol (mmol/L)	Unadjusted data	-	-	-	-
	Adjusted data	-	-	-	-
Serum triglycerides (mmol/L)	Unadjusted data	-	-	-	-
	Adjusted data	-	-	-	-
Serum Lp(a) (nmol/L)	Unadjusted data	-	-	-	-
	Adjusted data	-	-	-	-
Serum CRP (mg/L)	Unadjusted data	-	-	-	-
	Adjusted data	-	-	-	-
Plasma homocysteine (μmol/L)	Unadjusted data	-	-	-	-
	Adjusted data	-	-	-	-
Serum uric acid (μmol/L)	Unadjusted data	NTP vs PE w/SF	AUC 0.648, $p < 0.001$	28.82 % Criterion > 311.2 μmol/l	13.45% sensitivity at 97.5% specificity Criterion > 340.775 μmol/l (hyperuricemia)
	Adjusted data	-	-	-	-
BMI	Unadjusted data	NTP vs PE w/o SF	AUC 0.666, $p = 0.007$	20.00% Criterion > 27.78 (overweight)	20.0% sensitivity at 97.5% specificity Criterion > 31.02 (obese class I, moderately obese)
		NTP vs PE w/SF	AUC 0.672, $p < 0.001$	29.87% Criterion > 27.78 (overweight)	18.18% sensitivity at 97.5% specificity Criterion > 31.02 (obese class I, moderately obese)
	Adjusted data	NTP vs PE w/o SF	AUC 0.853, $p < 0.001$	62.50%	-

		NTP vs PE w/SF	AUC 0.781, $p < 0.001$	56.76%	-
Waist circumference (cm)	Unadjusted data	NTP vs PE w/o SF	AUC 0.702, $p = 0.001$	28.00%<br>Criterion > 87 cm	24.0% sensitivity at 91.11% specificity<br>Criterion > 88cm<br>(obese, high cardiovascular risk)
		NTP vs PE w/SF	AUC 0.683, $p < 0.001$	33.77%<br>Criterion > 87 cm	31.17% sensitivity at 91.11% specificity<br>Criterion > 88cm<br>(obese, high cardiovascular risk)
	Adjusted data	NTP vs PE w/o SF	AUC 0.847, $p < 0.001$	58.33%	-
		NTP vs PE w/SF	AUC 0.788, $p < 0.001$	55.41%	-
SBP (mmHg)	Unadjusted data	NTP vs PE w/o SF	AUC 0.740, $p < 0.001$	40.00%<br>Criterion > 123.4 mmHg<br>(prehypertension)	4.00% sensitivity at 100.0% specificity<br>Criterion > 141 mm Hg<br>(hypertension)
		NTP vs PE w/SF	AUC 0.753, $p < 0.001$	49.09%<br>Criterion > 123.4 mmHg<br>(prehypertension)	12.99% sensitivity at 100.0% specificity<br>Criterion > 141 mm Hg<br>(hypertension)
	Adjusted data	NTP vs PE w/o SF	AUC 0.805, $p < 0.001$	62.50%	-
		NTP vs PE w/SF	AUC 0.762, $p < 0.001$	56.76%	-
DBP (mmHg)	Unadjusted data	NTP vs PE w/o SF	AUC 0.669, $p = 0.002$	24.00%<br>Criterion > 80.5 mmHg<br>(prehypertension)	12.00% sensitivity at 100.0% specificity<br>Criterion > 91 mm Hg<br>(hypertension)
		NTP vs PE w/SF	AUC 0.729, $p < 0.001$	44.81%<br>Criterion > 80.5 mmHg<br>(prehypertension)	12.99% sensitivity at 100.0% specificity<br>Criterion > 91 mm Hg<br>(hypertension)
	Adjusted data	NTP vs PE w/o SF	AUC 0.729, $p < 0.001$	45.83%	-
		NTP vs PE w/SF	AUC 0.747, $p < 0.001$	50.00%	-
Heart rate (bpm)	Unadjusted data	-	-	-	-
	Adjusted data	-	-	-	-
Relative QRISK®2 risk score	Unadjusted data	NTP vs PE w/o SF	AUC 0.723, $p < 0.001$	19.87%<br>Criterion > 1.60	0.0% sensitivity at 100.0% specificity<br>Criterion > 2.9
		NTP vs PE w/SF	AUC 0.707, $p < 0.001$	28.48%	16.88% sensitivity at 100.0% specificity

			Criterion > 1.60	Criterion > 2.9
Adjusted data	NTP vs PE w/o SF	AUC 0.843, $p < 0.001$	58.33%	-
	NTP vs PE w/SF	AUC 0.782, $p < 0.001$	54.05%	-

The unadjusted and adjusted receivers operating characteristic (ROC) curves were constructed to calculate the area under the curve (AUC) and the best cut-off points for particular studied parameters or biomarkers were used in order to calculate the respective sensitivity at 90.0% specificity (MedCalc Software bvba, Ostend, Belgium). We also reported for unadjusted data the information showing the sensitivity at criterions exceeding the critical values (number of cases exceeding the critical values). Data were adjusted for potential covariates, where appropriate, including current maternal age, BMI, parity, the time after the birth (in months), the oral contraceptive use status (ex-user, current user, non-user), the smoking status (ex-smoker, smoker, non-smoker), the average values of systolic and diastolic blood pressures, and hypertension on the treatment (yes, no). The significance level was established at *p*-value of $p < 0.05$. PE w/o SF, preeclampsia without severe features; PE w/SF, preeclampsia with severe features; NTP, normotensive term pregnancies; HDL, high-density lipoprotein; LDL, low-density lipoprotein; Lp(a), lipoprotein a; CRP, C-reactive protein; BMI, body mass index; SBP, systolic blood pressure; DBP, diastolic blood pressure; bpm, beats per minute; AUC, area under curve; FPR, false positive rate.

Table S5. Impact of PE with respect to delivery date on maternal cardiovascular risk - complete overview (ANOVA and ANCOVA analyses).

		**NTP (*n* = 90)**	**Early PE (*n* = 36)**	**Late PE (*n* = 66)**	**Diagnostic Groups (Normal vs Diseased)**	***p* Value (ANOVA, ANCOVA)**
Serum total cholesterol (mmol/L)	Unadjusted data	5.040 (0.081)	5.173 (0.128)	5.160 (0.095)	-	-
	Adjusted data	5.128 (0.086)[A]	5.049 (0.142)[A]	5.131 (0.095)[A]	-	-
Serum HDL cholesterol (mmol/L)	Unadjusted data	1.606 (0.031)	1.519 (0.049)	1.535 (0.036)	-	-
	Adjusted data	1.574 (0.032)[A]	1.606 (0.053)[A]	1.541 (0.035)[A]	-	-
Serum LDL cholesterol (mmol/L)	Unadjusted data	3.119 (0.069)	3.318 (0.109)	3.254 (0.080)	-	-
	Adjusted data	3.211 (0.072)[A]	3.152 (0.120)[A]	3.220 (0.080)[A]	-	-
Serum triglycerides (mmol/l)	Unadjusted data	0.847 (0.050)	0.926 (0.079)	1.016 (0.058)	-	-
	Adjusted data	0.879 (0.054)[A]	0.891 (0.089)[A]	1.007 (0.060)[A]	-	-
Serum Lp(a) (nmol/L)	Unadjusted data	36.449 (7.509)	90.828 (11.974)	45.483 (8.720)	**NTP vs ↑early PE**	***p* = 0.037**
	Adjusted data	38.909 (8.094)[A]	88.341 (13.631)[A]	46.977 (8.921)[A]	NTP vs early PE	*p* = 0.192
Serum CRP (mg/L)	Unadjusted data	3.395 (0.870)	3.041 (1.368)	2.828 (1.010)	-	-
	Adjusted data	4.058 (0.948)[A]	1.565 (1.564)[A]	2.604 (1.043)[A]	-	-
Plasma homocysteine (µmol/L)	Unadjusted data	10.917 (0.311)	11.279 (0.496)	11.439 (0.361)	-	-
	Adjusted data	10.716 (0.338)[A]	11.716 (0.570)[A]	11.651 (0.373)[A]	-	-
Serum uric acid (µmol/L)	Unadjusted data	248.044 (5.625)	285.971 (8.971)	271.924 (6.532)	**NTP vs ↑early PE**<br>**NTP vs ↑late PE**	***p* = 0.001**<br>***p* = 0.018**
	Adjusted data	258.299 (5.694)[A]	266.431 (9.590)[A]	270.292 (6.276)[A]	-	-
BMI	Unadjusted data	23.100 (0.465)	28.085 (0.735)	24.779 (0.543)	**NTP vs ↑early PE**<br>**NTP vs ↑late PE**	***p* < 0.001**<br>***p* = 0.034**
	Adjusted data	23.440 (0.475)[A]	26.948 (0.784)[A]	24.998 (0.535)[A]	**NTP vs early PE**<br>**NTP vs ↑late PE**	***p* = 0.003**<br>***p* = 0.031**
Waist circumference (cm)	Unadjusted data	76.605 (1.161)	88.472 (1.836)	81.333 (1.356)	**NTP vs ↑early PE**<br>**NTP vs ↑late PE**	***p* < 0.001**<br>***p* = 0.006**
	Adjusted data	77.654 (1.202)[A]	85.201 (1.983)[A]	81.578 (1.353)[A]	**NTP vs ↑early PE**<br>**NTP vs ↑late PE**	***p* = 0.011**<br>***p* = 0.018**

SBP (mmHg)	Unadjusted data	112.911(1.178)	128.055 (1.864)	121.257 (1.376)	**NTP vs ↑early PE**<br>**NTP vs ↑late PE**	***p* < 0.001**<br>***p* < 0.001**
	Adjusted data	116.931 (0.746)[A]	120.196 (1.243)[A]	120.052 (0.845)[A]	NTP vs ↑early PE<br>**NTP vs ↑late PE**	*p* = 0.099<br>***p* = 0.021**
DBP (mmHg)	Unadjusted data	72.100 (0.914)	83.166 (1.446)	77.484 (1.068)	**NTP vs ↑early PE**<br>**NTP vs ↑late PE**	***p* < 0.001**<br>***p* < 0.001**
	Adjusted data	72.837 (0.937)[A]	81.848 (1.554)[A]	77.570 (1.091)[A]	**NTP vs ↑early PE**<br>**NTP vs ↑late PE**	***p* < 0.001**<br>***p* = 0.004**
Heart rate (bpm)	Unadjusted data	71.644 (1.024)	72.611 (1.620)	72.636 (1.196)	-	-
	Adjusted data	72.347 (1.083)[A]	71.673 (1.799)[A]	72.337 (1.199)[A]	-	-
Relative QRISK®2 risk score	Unadjusted data	0.920 (0.108)	2.436 (0.170)	1.171 (0.125)	**NTP vs ↑early PE**<br>**NTP vs ↑late PE**	***p* < 0.001**<br>***p* = 0.009**
	Adjusted data	1.036 (0.093)[A]	2.099 (0.151)[A]	1.162 (0.105)[A]	**NTP vs ↑early PE**<br>**NTP vs ↑late PE**	***p* < 0.001**<br>***p* = 0.005**

Data are expressed as mean (SE; standard error). Analysis of variance (ANOVA) was used for unadjusted data and Analysis of covariance (ANCOVA) for adjusted data. The significance level was established at *p*-value of $p < 0.05$ (Bonferroni corrected p-values). [A] Adjusted for potential covariates, where appropriate, including current maternal age, BMI, parity, the time after the birth (in months), the oral contraceptive use status (ex-user, current user, non-user), the smoking status (ex-smoker, smoker, non-smoker), the average values of systolic and diastolic blood pressures, and hypertension on the treatment (yes, no). PE, preeclampsia; NTP, normotensive term pregnancies; HDL, high-density lipoprotein; LDL, low-density lipoprotein; Lp(a), lipoprotein a; CRP, C-reactive protein; BMI, body mass index; SBP, systolic blood pressure; DBP, diastolic blood pressure; bpm, beats per minute.

Table S6. Impact of PE with respect to delivery date on maternal cardiovascular risk – complete overview (ROC curve analysis).

		Diagnostic Groups (Normal vs Diseased)	ROC Curve Parameters	Sensitivity at 10% FPR	Sensitivity and Specificity When Critical Values Are Exceeded
Serum total cholesterol (mmol/l)	Unadjusted data	-	-	-	-
	Adjusted data	-	-	-	-
Serum HDL cholesterol (mmol/l)	Unadjusted data	-	-	-	-
	Adjusted data	-	-	-	-
Serum LDL cholesterol (mmol/l)	Unadjusted data	-	-	-	-
	Adjusted data	-	-	-	-
Serum triglycerides (mmol/l)	Unadjusted data	-	-	-	-
	Adjusted data	-	-	-	-
Serum Lp(a) (nmol/l)	Unadjusted data	NTP vs early PE	AUC 0.632, $p = 0.022$	31.43% Criterion > 89.26 nmol/l (risk of CVD)	34.29% sensitivity at 86.52% specificity Criterion > 73.20 nmol/l (risk of CVD)
	Adjusted data	NTP vs early PE	AUC 0.905, $p < 0.001$	81.82 %	-
Serum CRP (mg/l)	Unadjusted data	-	-	-	-
	Adjusted data	-	-	-	-
Plasma homocysteine (μmol/l)	Unadjusted data	-	-	-	-
	Adjusted data	-	-	-	-
Serum uric acid (μmol/l)	Unadjusted data	NTP vs early PE	AUC 0.667, $p = 0.004$	42.86% Criterion > 308.5 μmol/l	20.00% sensitivity at 97.5% specificity Criterion > 340.77 μmol/l (hyperuricemia)
		NTP vs late PE	AUC 0.632, $p = 0.003$	20.91% Criterion > 311.2 μmol/l	9.43% sensitivity at 97.5% specificity Criterion > 340.55 μmol/l (hyperuricemia)
	Adjusted data	-	-	-	-
BMI	Unadjusted data	NTP vs early PE	AUC 0.748, $p < 0.001$	38.89% Criterion > 27.78	33.33% sensitivity at 97.5% specificity Criterion > 31.02

				(overweight)	(obese class I, moderately obese)
		NTP vs late PE	AUC 0.628, $p = 0.004$	21.21%<br>Criterion > 27.78<br>(overweight)	10.61% sensitivity at 97.5% specificity<br>Criterion > 31.02<br>(obese class I, moderately obese)
	Adjusted data	NTP vs early PE	AUC 0.894, $p < 0.001$	67.65%	-
		NTP vs late PE	AUC 0.767, $p < 0.001$	48.44%	-
Waist circumference (cm)	Unadjusted data	NTP vs early PE	AUC 0.740, $p < 0.001$	50.00%<br>Criterion > 87 cm	44.44% sensitivity at 91.11% specificity<br>Criterion > 88cm<br>(obese, high cardiovascular risk)
		NTP vs late PE	AUC 0.659, $p < 0.001$	22.73%<br>Criterion > 87 cm	21.21% sensitivity at 91.11% specificity<br>Criterion > 88cm<br>(obese, high cardiovascular risk)
	Adjusted data	NTP vs early PE	AUC 0.890, $p < 0.001$	64.71%	-
		NTP vs late PE	AUC 0.775, $p < 0.001$	50.00%	-
SBP (mmHg)	Unadjusted data	NTP vs early PE	AUC 0.859, $p < 0.001$	62.78%<br>Criterion > 123.4 mmHg<br>(prehypertension)	16.67 % sensitivity at 100.0% specificity<br>Criterion > 141 mmHg<br>(hypertension)
		NTP vs late PE	AUC 0.690, $p < 0.001$	38.18%<br>Criterion > 123.4 mmHg<br>(prehypertension)	7.58 % sensitivity at 100.0% specificity<br>Criterion > 141 mmHg<br>(hypertension)
	Adjusted data	NTP vs early PE	AUC 0.884, $p < 0.001$	70.59%	-
		NTP vs late PE	AUC 0.724, $p < 0.001$	46.88%	-
DBP (mmHg)	Unadjusted data	NTP vs early PE	AUC 0.824, $p < 0.001$	54.17%<br>Criterion > 80.5 mmHg<br>(prehypertension)	16.67 % sensitivity at 100.0% specificity<br>Criterion > 91 mmHg<br>(hypertension)
		NTP vs late PE	AUC 0.654, $p < 0.001$	31.82%<br>Criterion > 80.5 mmHg<br>(prehypertension)	10.61 % sensitivity at 100.0% specificity<br>Criterion > 91 mmHg<br>(hypertension)
	Adjusted data	NTP vs early PE	AUC 0.874, $p < 0.001$	64.71%	-
		NTP vs late PE	AUC 0.704, $p < 0.001$	34.38%	-

Heart rate (bmp)	Unadjusted data	-	-	-	-
	Adjusted data	-	-	-	-
Relative QRISK®2 risk score	Unadjusted data	NTP vs early PE	AUC 0.802, $p < 0.001$	41.67% Criterion > 1.60	33.33% sensitivity at 100.0% specificity Criterion > 2.9
		NTP vs late PE	AUC 0.661, $p < 0.001$	18.03% Criterion > 1.60	1.52% sensitivity at 100.0% specificity Criterion > 2.9
	Adjusted data	NTP vs early PE	AUC 0.886, $p < 0.001$	73.53%	-
		NTP vs late PE	AUC 0.749, $p < 0.001$	51.56%	-

The unadjusted and adjusted receivers operating characteristic (ROC) curves were constructed to calculate the area under the curve (AUC) and the best cut-off points for particular studied parameters or biomarkers were used in order to calculate the respective sensitivity at 90.0% specificity (MedCalc Software bvba, Ostend, Belgium). We also reported for unadjusted data the information showing the sensitivity at criterions exceeding the critical values (number of cases exceeding the critical values). Data were adjusted for potential covariates, where appropriate, including current maternal age, BMI, parity, the time after the birth (in months), the oral contraceptive use status (ex-user, current user, non-user), the smoking status (ex-smoker, smoker, non-smoker), the average values of systolic and diastolic blood pressures, and hypertension on the treatment (yes, no). The significance level was established at *p*-value of $p < 0.05$. PE, preeclampsia; NTP, normotensive term pregnancies; HDL, high-density lipoprotein; LDL, low-density lipoprotein; Lp(a), lipoprotein a; CRP, C-reactive protein; BMI, body mass index; SBP, systolic blood pressure; DBP, diastolic blood pressure; bpm, beats per minute; AUC, area under curve; FPR, false positive rate. .
